# Dietary inflammatory potential is associated with higher odds of hepatic steatosis in US adults: a cross-sectional study

**DOI:** 10.1017/S1368980023001970

**Published:** 2023-12

**Authors:** Hu Yang, Tengfei Zhang, Wen Song, Zhaohong Peng, Yu Zhu, Yong Huang, Xiude Li, Zhuang Zhang, Min Tang, Wanshui Yang

**Affiliations:** 1 Department of Nutrition, School of Public Health, Anhui Medical University, 81 Meishan Road, Hefei, Anhui 230032, People’s Republic of China; 2 Taizhou Central Hospital (Taizhou University Hospital), Taizhou, Zhejiang, People’s Republic of China; 3 Department of Interventional Radiology, the First Affiliated Hospital of Anhui Medical University, Hefei, Anhui, People’s Republic of China; 4 Department of Gastroenterology and Hepatology and Clinical Nutrition, the Fourth Affiliated Hospital of Anhui Medical University, Hefei, Anhui, People’s Republic of China

**Keywords:** Cross-sectional study, Diet, Inflammation, Hepatic steatosis, Controlled attenuation parameter

## Abstract

**Objective:**

Inflammation plays a critical role in the progression of chronic liver diseases, and diet can modulate inflammation. Whether an inflammatory dietary pattern is associated with higher risk of hepatic steatosis or fibrosis remains unclear. We aimed to investigate the associations between inflammatory dietary pattern and the odds of hepatic steatosis and fibrosis.

**Design:**

In this nationwide cross-sectional study, diet was measured using two 24-h dietary recalls. Empirical dietary inflammatory pattern (EDIP) score was derived to assess the inflammatory potential of usual diet, which has been validated to highly predict inflammation markers in the study population. Controlled attenuation parameter (CAP) and liver stiffness measurement (LSM) were derived from FibroScan to define steatosis and fibrosis, respectively.

**Setting:**

US National Health and Nutrition Examination Survey.

**Participants:**

4171 participants aged ≥18 years.

**Results:**

A total of 1436 participants were diagnosed with S1 steatosis (CAP ≥ 274 dB/m), 255 with advanced fibrosis (LSM ≥ 9·7 kPa). Compared with those in the lowest tertile of EDIP-adherence scores, participants in the highest tertile had 74 % higher odds of steatosis (OR: 1·74, 95 % CI (1·26, 2·41)). Such positive association persisted among never drinkers, or participants who were free of hepatitis B and/or C. Similarly, EDIP was positively associated with CAP in multivariate linear model (*P* < 0·001). We found a non-significant association of EDIP score with advanced fibrosis or LSM (*P* = 0·837).

**Conclusions:**

Our findings suggest that a diet score that is associated with inflammatory markers is associated with hepatic steatosis. Reducing or avoiding pro-inflammatory diets intake might be an attractive strategy for fatty liver disease prevention.

Non-alcoholic fatty liver disease (NAFLD) imposes an enormous burden on health care systems and affects approximately 25 % of the population worldwide and 30 % of people in the USA^(1)^. To date, due to the lack of approved drug therapy, lifestyle modification to achieve weight loss remains an optimal intervention for patients with NAFLD^(1,2)^. Accumulating evidence indicates that chronic inflammation contributes substantially to NAFLD pathogenesis^(3)^. Circulating concentrations of inflammation markers, such as IL-4, IL-6, C-reactive protein (CRP) and tumour necrosis factor-*α* receptor 2 (TNF*α*-R2), have been shown to be associated with NAFLD in prior studies^(1,4,5)^. Moreover, in previous studies, lifestyles including diets can modulate inflammation^(6–10)^. For instance, Mediterranean-type diets have anti-inflammatory properties and are effective in decreasing the risk of NAFLD and slowing its progression^(11)^. Thus, we hypothesised that higher inflammatory potential of diet might be associated with increased risk of hepatic steatosis or fibrosis.

Recently, we re-derived and validated an empirical dietary inflammatory pattern (EDIP) in the US National Health and Nutrition Examination Survey (NHANES)^(12)^, which is originally developed by Tabung *et al.* in three Harvard cohorts^(13)^. EDIP is a hypothesis-driven *a posteriori* dietary pattern and was derived by entering thirty-nine predefined food groups into the reduced rank regression (RRR) followed by stepwise linear regression, which was highly predictive of concentration of two plasma inflammation markers including CRP and leucocytes count. EDIP has been suggested to be associated with higher risk of several chronic diseases including CVD^(14)^, cancers^(15–17)^ and type 2 diabetes^(18)^. Moreover, we previously showed that EDIP is positively associated with risk of total and cancer-specific mortality^(12)^, and hepatocellular carcinoma^(16)^. However, to our knowledge, there have been no epidemiological studies regarding the association between EDIP and hepatic steatosis and fibrosis to date, although few studies^(19–22)^ have investigated hepatic steatosis in relation to a literature-derived dietary inflammatory index (DII), which is an *a priori* dietary pattern (i.e. its development is based on the peer-reviewed articles on the association between dietary factors and inflammation). Given that DII is mainly nutrient-based (i.e. thirty-eight of its forty-five components are nutrients), findings from DII studies could be difficult to be translated readily into public health practice.

Herein, to add more evidence, we investigated the cross-sectional association between adherence to EDIP and odds of hepatic steatosis and fibrosis in a US nationwide sample.

## Methods

### Study population

This study used data from the 2017–2018 cycle of the US NHANES, in which hepatic transient elastography (TE) was performed for the first time in the survey. NHANES is a continuous cross-sectional survey conducted in the USA by the National Center for Health Statistics of the Centers for Disease Control and Prevention. The survey aimed to assess the health of a representative sample of about 5000 persons each year in the USA. Details of NHANES study design, study protocol and data collection approaches have previously been reported^(23)^.

The flow chart of how we selected the study population was shown in Supplemental Fig. 1. Individuals aged 18 years or older were included. We excluded participants if they: (i) had missing data on diet (*n* 873); (ii) had implausible energy intake^(16)^ (<600 or >3500 kcal/d for women; <800 or >4200 kcal/d for men, *n* 221); (iii) did not receive TE detection (*n* 264) or (iv) had invalid TE detection results (*n* 327). A total of 4171 participants were finally included in the analysis.

### Assessments of diet and empirical dietary inflammatory pattern score

Dietary information was collected using two 24-h dietary recalls by skilled investigators. We used multiple-pass method to enhance complete and accurate data collection and decrease respondent burden^(24)^. Dietary sampling weights were used to overcome the limitations including the dietary interview-specific nonresponse, day of the week for dietary recalls, unequal probability of selection and oversampling^(24,25)^.

The development and validation of EDIP scores have been described previously^(12)^. In short, thirty-nine pre-defined food groups^(26)^ were entered into RRR model followed by stepwise linear regression analysis to identify a dietary pattern most predictive of two inflammation markers (i.e. CRP and leucocytes count). RRR can identify linear functions of predictors (i.e. food groups) that simultaneously explain as much response variation of inflammation markers as possible. The first factor (i.e. the RRR dietary pattern) identified by RRR then underwent further data reduction by stepwise linear regression to identify the most important component food groups of the RRR dietary pattern, with the RRR dietary pattern as the dependent variable, the thirty-nine food groups as independent variables, and a significance level of *P* = 0·01 for entry into, and retention in the model. A total of twenty-five food components were included in EDIP score (see online Supplemental Table 1). We used the regression coefficients in the final stepwise linear regression model as weights to calculate the EDIP scores. Higher EDIP scores (more positive) denote more pro-inflammatory potential of diets, while lower (more negative) scores indicate anti-inflammatory potential of diets.

In validation study of our prior publication^(12)^, EDIP have shown a high ability to predict inflammatory markers (i.e. plasma high-sensitivity CRP and leucocytes count) in NHANES 2015–2018.

### Assessments of covariates

Information on demographic and lifestyle factors, including age, sex, race/ethnicity, educational level, income, smoking and physical activity, were collected by standardised questionnaires during household interview. Information on alcohol intake, body weight and height was obtained from participants who received physical examinations in the NHANES Mobile Examination Center. Individuals who had smoked at least 100 cigarettes in life were defined as ever smokers, and never smokers were defined as those who did not have cigarettes consumption before the time of the interview. BMI was calculated as weight in kilograms divided by the square of the height in meters (kg/m^2^). The ratio of family income to poverty that accounts for family size and annual inflation and was calculated by dividing family income by the poverty thresholds. The poverty thresholds were defined as the dollar amounts set by the U.S. government to indicate the least amount of income a person or family needs to meet their basic needs, which were used to estimate the population’s income and poverty levels and related information. Physical activity was assessed by Global Physical Activity Questionnaire, which has been shown to have good reliability and validity in multiple populations^(27)^. Individuals who performed <8·3, 8·3 to 16·7 and >16·7 metabolic equivalents of tasks hours of physical activity/week were classified as low, moderate and high levels according to the 2018 Physical Activity Guidelines for Americans^(28)^. Hepatitis B virus (HBV) infection was defined as positive hepatitis B surface antigen (HBsAg), while both positive hepatitis C antibody and RNA indicated hepatitis C virus (HCV) infection. Diabetes was diagnosed if there were: (i) a self-reported history of diabetes; (ii) a fasting plasma glucose level of more than 126 mg/dl; (iii) a random glucose level of more than 200 mg/dl and (iv) a HbA1c level of more than 6·5 % for participants.

### Ascertainments of hepatic steatosis and fibrosis

Vibration-controlled TE using the FibroScan® model 502 V2 Touch equipped with a medium (M) or extra-large (XL) wand (probe) was performed by technicians after a 2-d training program with an expert technician. Hepatic steatosis and fibrosis were measured using controlled attenuation parameter (CAP) and liver stiffness measurement (LSM), respectively. In accordance with previous studies^(29–31)^, we used cut-off values of median CAP ≥ 274 for S1 steatosis, CAP ≥ 290 for S2 steatosis, LSM ≥ 8·2 kPa for significant fibrosis and LSM ≥ 9·7 kPa for advanced fibrosis. TE examinations were considered as reliable only when more than 10 LSMs were obtained after a fasting time no less than 3 h, with an interquartile range (IQR) to median ratio < 30 %.

By comparing CAP measurement for the detection of steatosis against biopsy, the area under the receiver operating characteristic curves was 0·87 (95 % CI (0·82, 0·92)) with a sensitivity and specificity of both 90 % for S ≥ S1 among patients with NAFLD^(29)^. Similarly, when using LSM to define patients with fibrosis, the area under the receiver operating characteristic curves was 0·80 (95 % CI (0·75, 0·84)) for advanced fibrosis (F ≥ F3), with the corresponding sensitivity of 71 % and specificity of 75 %^(29)^.

### Statistical analysis

The prevalence of hepatic steatosis and fibrosis was standardised based on 2020 US population^(31)^. Multiple linear regression was performed to evaluate the percentage change and 95 % CI for the associations of the EDIP scores with continuous CAP and LSM. Both CAP and LSM were natural logarithms transformed in the models given the deviation from normal distribution. We used multiple logistic regression to estimate the OR and 95 % CI for EDIP scores in relation to S1 and S2 steatosis. Covariates adjusted in the models were as follows: Model 1 was adjusted for age (18–39, 40–59 and ≥60). Model 2 was further adjusted for sex (male and female), smoking status (never smokers and ever smokers), race/ethnicity (non-Hispanic white, non-Hispanic black and other races), education (less than high school, high school diploma and more than high school), family income to poverty ratio (<1·30, 1·30–3·49 and ≥3·50), marital status (never married, married and widowed/divorced), physical activity (low level, moderate level and high level), total energy intake (tertiles), HBV (positive and negative) and HCV (positive and negative) infection. Of note, we did not adjust for alcohol consumption in our main analyses because wine and beer are components in EDIP. For covariates with missing values, a separate missing indicator variable was created and included in the models. We presented OR by tertile categories and per 1-sd increase of EDIP scores. Linear trends across increasing categories of EDIP scores were tested by entering EDIP scores as a continuous variable in the models, and *P* values for trend were calculated using a Wald test. We also used restricted cubic spline to identify the dose–response relationship between EDIP and hepatic steatosis.

Allowing for the potential intermediate role of BMI and diabetes in the association of EDIP and chronic liver disease^(16)^, we did not adjust for BMI and diabetes in the main analyses but additionally adjusted for these two factors in the sensitivity analyses. To reduce measurement error and reflect dietary composition, we adjusted the EDIP scores for total energy intake using the nutrient residual method^(32)^. Considering that HBV and HCV infections are important risk factors for liver diseases, we repeated analysis within individuals who are free of hepatitis B and/or C. Likewise, we investigated EDIP after removing their alcohol components (i.e. beer and wine) in relation to the prevalence of hepatic steatosis, with further adjustments for alcohol drinking status (never, low to moderate and heavy drinking), although beer and wine are included in the construct of EDIP. In addition, considering the possible etiological differences, we investigated the association of EDIP with non-alcoholic fatty liver and other steatosis separately. The non-alcoholic fatty liver diseases were defined if individuals: (i) were detected as steatosis through TE test; (ii) did not have significant alcohol consumption (>2 drinks/d for women and >3 drinks/d for men); (iii) were free of hepatitis B and/or C infection and (iv) did not take steatogenic medications (i.e. amiodarone, valproate, methotrexate, tamoxifen and corticosteroid) for at least 3 months or more before study enrollment^(33,34)^.

Previous studies suggested that several factors including age, sex, race, smoking status, drinking status, marital status, BMI and diabetes could modify the associations between inflammatory dietary pattern and chronic liver diseases^(16,35–37)^. Therefore, we stratified analyses according to these factors and tested the potential interactions. Wald test was used to check whether the cross-product terms between these variables and exposures were statistically significant. We used the Bonferroni correction to define the statistical significance as *P* < 0·0031 (0·05/(2 outcomes x 8 groups)) for subgroup analysis to account for multiple comparisons. All statistical tests were two-tailed and performed using SAS version 9.4 (SAS Institute).

## Results

### Characteristics of participants

A total of 4171 participants aged 18 years or older (mean age, 49·4 years; sd, 18·3 years) were included in our study. The age-standardised prevalence was 42·5 % (1806 cases) for S1 steatosis, 33·8 % (1436 cases) for S2 steatosis and 5·6 % (255 cases) for advanced fibrosis. The median (IQR) of EDIP scores for the total population was −0·03 (IQR: −0·15 to 0·06), ranged from a median of −0·21 (IQR: −0·31 to −0·15) in the lowest tertile to 0·09 (IQR: 0·06 to 0·15) in the highest tertile. Participants with higher EDIP score were older, had higher BMI, were more likely to be ever smokers and non-Hispanic whites, were less educated, were more likely to be married, had lower ratio of family income to poverty, were less physically active and were more likely to have a history of diabetes and hepatitis C (Table 1).

**Table 1:** Age-adjusted characteristics of participants according to tertiles of EDIP scores in NHANES 2017–2018*

Characteristics	EDIP scores					
	Tertile 1 (*n* 1390)		Tertile 2 (*n* 1391)		Tertile 3 (*n* 1390)	
	Mean	IQR	Mean	IQR	Mean	IQR
EDIP scores†	−0·21	−0·31, −0·15	−0·03	−0·07, 0·00	0·09	0·06, 0·15
	Mean	sd	Mean	sd	Mean	sd
Age (years)†	48·1	17·7	48·3	18·6	51·8	18·2
BMI (kg/m^2^)	28·8	6·8	29·6	7·1	29·9	7·2
METS-h/week||	78·1	124·9	63·7	103·7	78·8	124·7
Energy (kcal/d)	2295	708	1858	640	1804	686
CAP (dB/m)	259·2	61·8	263·7	61·7	268·2	64·3
LSM (kPa)	5·6	4·1	5·6	3·8	6·0	5·3
	*n*	%	*n*	%	*n*	%
Male (%)	749	53·9	618	44·4	666	47·9
Drinking status (%)						
Never	143	10·3	160	11·5	154	11·1
Low to moderate‡	1049	75·5	1085	78·0	1090	78·4
Heavy	198	14·1	146	10·5	146	10·5
Ever smokers (%)§	489	35·2	522	37·5	674	48·5
Race (%)						
Non-Hispanic white	407	29·3	408	29·3	645	46·4
Non-Hispanic black	363	26·1	374	26·9	214	15·4
Other	620	44·6	609	43·7	531	38·2
Education (%)						
Less than high school	202	14·5	256	18·4	317	22·8
High school diploma	278	20·0	345	24·8	413	29·7
More than high school	910	65·5	790	56·8	660	47·5
Family income to poverty ratio (%)						
<1·30	318	22·9	417	30·0	453	32·6
1·30–3·49	539	38·8	551	39·6	580	41·7
≥3·50	533	38·3	423	30·4	357	25·7
Marital status (%)						
Never married	374	26·9	391	28·1	370	26·6
Married	774	55·7	762	54·8	733	52·7
Widowed or divorced	242	17·4	238	17·1	287	20·7
Physical activity (%)						
Low level	434	31·2	505	36·3	510	36·7
Moderate level	145	10·4	145	10·4	114	8·2
High level	811	58·4	741	53·3	766	55·1
Diabetes (%)	232	16·7	273	19·6	278	20·0
HBV infection (%)	10	0·75	2	0·15	6	0·41
HCV infection (%)	14	1·04	22	1·58	41	2·96

EDIP, empirical dietary inflammatory pattern; HBV, hepatitis B Virus; HCV, hepatitis C Virus; NHANES, US National Health and Nutrition Examination Survey.

* Continuous variables were presented as means (sd) if they were normally distributed, otherwise median (IQR) estimate was used; All the variables were standardised to the age distribution of the study population except for EDIP scores and age; Notably, the summing proportions for some categories are not 100 % because of missing values or rounding.

† Value was not age adjusted.

‡ Individuals who never drank in the last year but reported a history of alcohol drinking previously were also assigned in this category.

§ Individuals who had smoked at least 100 cigarettes in life.

|| Individuals who performed <8·3, 8·3 to 16·7, >16·7 METS-hours of physical activity/week were classified as low, medium, and high levels.

### EDIP, hepatic steatosis and fibrosis

After adjusting for age, sex, and other covariates (Table 2), EDIP score was positively associated with CAP, with the percentage difference of 7·4 % (95 % CI (4·1, 10·9), *P* < 0*·*001) in participants with the highest tertile of EDIP scores, compared with those in the lowest tertile. This positive association was partly attenuated but remained statistically significant with further adjustments for BMI and diabetes (percentage difference: 3·5 %, 95 % CI (1·7, 5·4), *P* < 0*·*001). We found a non-significant association between EDIP score and LSM (percentage difference: 5·4 %, 95 % CI (−0·4 , 11·5), *P =* 0·837).

**Table 2:** Percentage change (%) and 95 % CI for the associations of the empirical dietary inflammatory pattern with controlled attenuation parameter (CAP) and liver stiffness measurement (LSM) in NHANES (2017–2018)*

	Tertile 1	Tertile 2		Tertile 3		Per 1-sd		*P*_value_ †
		%	95 % CI	%	95 % CI	%	95 % CI	
CAP (dB/m)								
No. of participants	1390	1391		1390				
Model 1	Reference	2·6	−0·2, 5·6	5·7	2·2, 9·2	2·5	1·2, 3·8	<0·001
Model 2	Reference	4·2	1·6, 6·9	7·4	4·1, 10·9	3·1	2·0, 4·3	<0·001
LSM (kPa)								
Model 1	Reference	1·7	−4·9, 8·7	5·0	−0·4, 10·7	0·6	−2·1, 3·4	0·640
Model 2	Reference	3·6	−2·8, 10·5	5·4	−0·4, 11·5	0·3	−2·9, 3·6	0·837

EDIP, empirical dietary inflammatory pattern; HBV, hepatitis B Virus; HCV, hepatitis C Virus; NHANES, US National Health and Nutrition Examination Survey.

* Model 1 was adjusted for age; Model 2 was further adjusted for sex, smoking status, race, education, family income to poverty ratio, marital status, physical activity, total energy, HBV, and HCV.

† Linear trends across increasing categories of EDIP scores were tested by entering EDIP scores as a continuous variable into the models, and *P* values for trend were calculated using a Wald test.

**Fig. 1:**
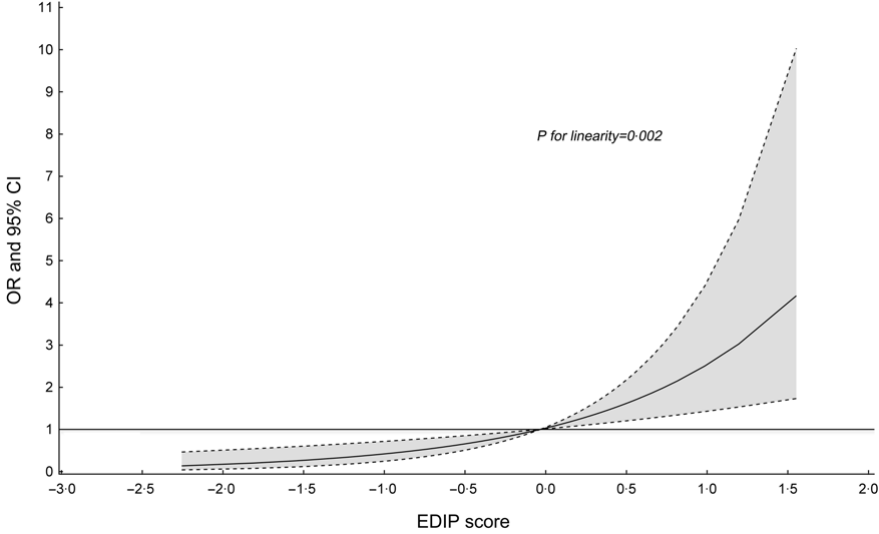
Association between empirical dietary inflammatory pattern scores and hepatic steatosis (≥S1) in NHANES (2017–2018)*. *Model was adjusted for age, sex, smoking status, race, education, family income to poverty ratio, marital status, physical activity, total energy, HBV, HCV, BMI and diabetes except for variables examined in the figure. Notably, the restricted multivariable cubic spline analysis showed significantly linear association between empirical dietary inflammatory pattern scores and hepatic steatosis (≥S1) (*P* for linearity = 0·002 and *P* for non-linearity = 0·157). Reference levels were set to the median EDIP value. Solid lines indicate OR, and dashed lines depict 95 % CI. EDIP, empirical dietary inflammatory pattern; HBV, hepatitis B Virus; HCV, hepatitis C Virus; NHANES, US National Health and Nutrition Examination Survey

Similarly, participants with higher EDIP score had higher odds of hepatic steatosis with OR (comparing extreme tertile) of 1·74 (95 % CI (1·26, 2·41), *P*_trend_ < 0*·*001, Table 3). When we additionally controlling BMI and diabetes, the magnitude of the positive association between EDIP and steatosis was partly attenuated (OR: 1·35, 95 % CI (1·04, 1·74), *P*_trend_ *=* 0*·*001). Similar association was observed with the cut-off value of median CAP of no less than 290 dB/m (OR: 1·34, 95 % CI (1·06, 1·70), *P*_trend_ *=* 0*·*004). Restricted cubic spline analysis did not support the non-linear association between EDIP and steatosis (*P* for linearity = 0*·*002, Fig. 1). We did not find any significant association between EDIP score and odds of significant fibrosis (LSM ≥ 8·2 kPa), advanced fibrosis (LSM ≥ 9·7 kPa) or cirrhosis (LSM ≥ 13·6 kPa, data not shown).

**Table 3:** Odds ratios and 95 % confidence intervals for hepatic steatosis according to tertiles of EDIP scores in NHANES 2017–2018*

	Tertile 1	Tertile 2		Tertile 3		Per 1-sd		*P*_trend_ ‡
		OR	95 % CI	OR	95 % CI	OR	95 % CI	
Steatosis† (≥S1)								
No. of cases	565	596		645				
Model 1	Reference	1·21	0·95, 1·55	1·47	1·09, 1·98	1·23	1·10, 1·38	<0·001
Model 2	Reference	1·36	1·09, 1·69	1·74	1·26, 2·41	1·33	1·16, 1·53	<0·001
Steatosis (≥S2)								
No. of cases	438	468		530				
Model 1	Reference	1·20	0·91, 1·57	1·52	1·16, 1·99	1·24	1·10, 1·39	<0·001
Model 2	Reference	1·33	1·04, 1·69	1·74	1·29, 2·34	1·32	1·14, 1·51	<0·001

EDIP, empirical dietary inflammatory pattern; HBV, hepatitis B Virus; HCV, hepatitis C Virus; NHANES, US National Health and Nutrition Examination Survey.

* Model 1 was adjusted for age; Model 2 was further adjusted for sex, smoking status, race, education, family income to poverty ratio, marital status, physical activity, total energy, HBV, and HCV.

† CAP values ≥ 274 dB/m and 290 dB/m were considered indicative of S1 and S2 steatosis, respectively.

‡ Linear trends across increasing categories of EDIP scores were tested by entering EDIP scores as a continuous variable into the models, and *P* values for trend were calculated using a Wald test.

### Sensitivity and subgroup analyses

**Fig. 2:**
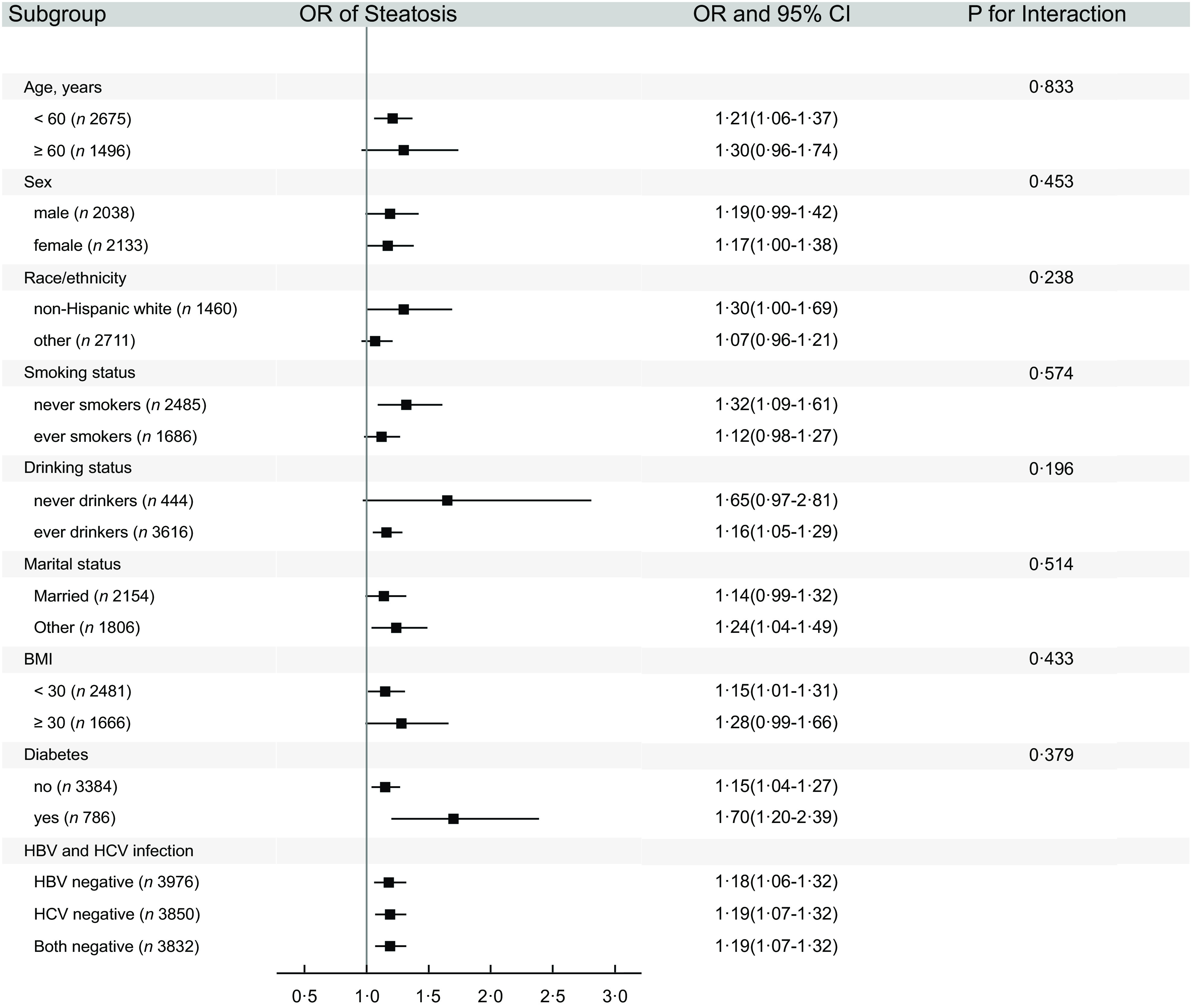
Subgroup analysis on the association of EDIP scores (per 1-sd increase) with hepatic steatosis (≥S1) in NHANES (2017–2018)*. *Model was adjusted for age, sex, smoking status, race, education, family income-poverty ratio, marital status, physical activity, total energy, HBV, HCV, BMI and diabetes except for variables examined in the figure. EDIP, empirical dietary inflammatory pattern; HBV, hepatitis B Virus; HCV, hepatitis C Virus; NHANES, US National Health and Nutrition Examination Survey

In sensitivity analysis, when repeated analysis using the energy-adjusted EDIP scores, the results were similar to those in the main analysis (see online Supplemental Table 2). Likewise, the results were not essentially changed among individuals who were free of hepatitis B and/or C (Fig. 2). After removing alcohol components in EDIP and further adjusted for alcohol intake in the models, the results were essentially unchanged (see online Supplemental Table 3). When examining non-alcoholic fatty liver and other steatosis separately, we did not find the significant heterogeneity on the associations of EDIP with odds of non-alcoholic fatty liver and other steatosis (*P*_heterogeneity_ = 0·477) (see online Supplemental Table 4).

In subgroup analysis (Fig. 2), there was no differential association between EDIP and odds of hepatic steatosis when stratified by age, sex, race/ethnicity, smoking status, drinking status, marital status, BMI or diabetes (all the *P* values for interaction were greater than Bonferroni-corrected statistical significance of 0·0031).

## Discussion

In this nationwide cross-sectional study among US adults, we examined associations between EDIP and odds of hepatic steatosis and fibrosis. We found that persons with higher EDIP scores (i.e. consuming a pro-inflammatory diet) had a higher prevalence of hepatic steatosis. This positive association remained among individuals who were free of hepatitis B and/or C and persisted regardless of alcohol drinking status. EDIP seemed not to be associated with fibrosis, as indicated by LSM.

Previous studies have reported that several nutrients and foods, such as fructose^(38,39)^, soft drinks^(40)^ and red meat^(40)^, have been associated with high risk for NAFLD. However, diets are complex combinations of nutrients and foods, which may interact mutually^(41,42)^. Thus, dietary patterns considering multiple dietary factors may provide a more comprehensive assessment of diet and may thus be more predictive of diet–disease associations compared with the approach of using single nutrients or foods.

This is the first observational study to investigate the association of EDIP score with hepatic steatosis and fibrosis among the US adults, though few studies have assessed the association between DII and fatty liver diseases or their parameters, which all used cross-sectional design^(19,21,22)^. The EDIP and DII both evaluate the inflammation potential of diet, while the two dietary patterns differ in several aspects. The EDIP is a hypothesis-driven *a posteriori* pattern (i.e. its development is based on RRR to identify food groups predictive of inflammation biomarkers) and is based exclusively on food groups. The DII is an *a priori* pattern (i.e. its development is based on the 1943 peer-reviewed articles on the association between dietary factors and inflammation) and is mainly nutrient-based. Different from our study, previous DII studies on fatty liver diseases used different approaches for outcome ascertainment, including Fatty Liver Index^(19,21,22)^, the aspartate transaminase to alanine transaminase ratio^(19)^ or fibrosis-4 score^(19)^, with the exception of 2 studies^(19,43)^. In the current study, we were able to derive CAP and LSM through TE (FibroScan®) to define hepatic steatosis and fibrosis with higher sensitivity and specificity^(29,44)^. However, our study together with previous DII studies^(19,21,22,43)^ consistently support that pro-inflammatory diets are associated with higher risk of fatty liver diseases.

In line with our study and previous DII studies^(19,21,22,43)^, a randomised controlled trial^(20)^ among younger adults with obesity showed the effectiveness of an energy-reduced anti-inflammatory diet with significant improvement of liver parameters, including Fatty Liver Index, liver fat score and fibrosis-4. Consistently, in two Harvard cohorts, the Nurses’ Health Study and the Health Professionals Follow-up Study of 119 316 participants with 142 incident hepatocellular carcinoma cases, we found a positive association between EDIP score and risk of hepatocellular carcinoma^(16)^. These findings further support that a diet score that is associated with inflammatory markers is associated with hepatic steatosis.

The association between greater adherence to pro-inflammatory diet and higher odds of hepatic steatosis has its biological plausibility. It is accepted that insulin resistance is a crucial pathophysiological factor in the development of NAFLD^(45,46)^, since the decreased insulin sensitivity of adipocyte causes an increased hepatic-free fatty acid flux creating favourable conditions for the progression of hepatic steatosis^(46,47)^. Moreover, inflammation cytokines, such as IL and TNFα, may disrupt insulin action and mediate insulin resistance^(24,48,49)^. Meantime, the elevated levels of inflammatory markers (i.e. CRP, IL-6 and TNF*α*) are observed among individuals with NAFLD^(50,51)^. Thus, one possible mechanism is that diet can modulate inflammation and mediate insulin resistance, which in turn leads to hepatic steatosis. However, we did not find any significant association between EDIP-adherence score and the likelihood of fibrosis (data not shown). One possible reason is that coffee is included as a component in EDIP (see online Supplemental Table 1), whereas coffee could induce UDP glucuronosyltransferases, which may contribute to the protective, antioxidant effects in the progression of hepatic fibrosis^(52–54)^. Alternatively, the lack of an association between EDIP and fibrosis may be due to the insufficient power caused by limited cases of fibrosis in the present study.

Strengths of our study include the use of validated food-based EDIP scores, a large nationally representative sample of US adults and valid TE detection to measure hepatic steatosis and fibrosis. However, our study has several limitations. First, dietary information was measured by 24-h recalls, which may limit the ability to capture habitual diets of individuals. To overcome this limitation, we used several methods, such as multiple-pass method and dietary sampling weights^(24,25)^. We also used energy-adjusted EDIP in the models^(32)^ and yielded similar results. Second, we are unable to completely rule out residual or unmeasured confounders (e.g. the use of anti-inflammation drugs). Third, the cross-sectional design in the current study does not allow the determination of causality.

In conclusion, findings from our study indicate that a diet score that is associated with inflammatory markers is associated with hepatic steatosis. Interventions to reduce the adverse effect of pro-inflammatory diet may reduce the likelihood of hepatic steatosis among US adults. However, our results should be interpreted with caution, given the measurement of diet using 24-h recalls and the cross-sectional design in the current study. More prospective cohort studies and clinical trials are needed to validate our findings.

## Supplementary material

For supplementary material accompanying this paper visit https://doi.org/10.1017/S1368980023001970
